# Natural Progression of Non-Alcoholic Steatohepatitis to Hepatocellular Carcinoma

**DOI:** 10.3390/biomedicines9020184

**Published:** 2021-02-12

**Authors:** Daryl Ramai, Waqqas Tai, Michelle Rivera, Antonio Facciorusso, Nicola Tartaglia, Mario Pacilli, Antonio Ambrosi, Christian Cotsoglou, Rodolfo Sacco

**Affiliations:** 1 Department of Internal Medicine, The Brooklyn Hospital Center, Brooklyn, NY 11201, USA; dramai@tbh.org (D.R.); wtai@tbh.org (W.T.); mirivera@tbh.org (M.R.); 2 Section of Gastroenterology, Department of Medical and Surgical Sciences, University of Foggia, 71122 Foggia, Italy; antonio.facciorusso@virgilio.it; 3 General Surgery Unit, Department of Medical and Surgical Sciences, University of Foggia, 71122 Foggia, Italy; nicola.tartaglia@unifg.it (N.T.); m.pacilli2010@gmail.com (M.P.); antonio.ambrosi@unifg.it (A.A.); 4 General Surgey Unit, Department of Surgery, ASST-Vimercate, 20871 Vimercate, Italy; christian.cotsoglou@asst-vimercate.it

**Keywords:** non-alcoholic steatohepatitis, hepatocellular carcinoma, pathogenesis

## Abstract

Non-alcoholic steatohepatitis (NASH) is a chronic and progressive form of non-alcoholic fatty liver disease (NAFLD). Its global incidence is increasing which makes NASH an epidemic and a public health threat. Due to repeated insults to the liver, patients are at risk for developing hepatocellular carcinoma (HCC). The progression of NASH to HCC was initially defined according to a two-hit model which involved the development of steatosis, followed by lipid peroxidation and inflammation. However, current research defines a “multi-hit” or “multi-parallel hit” model which synthesizes several contributing pathways involved in progressive fibrosis and oncogenesis. This perspective considers the effects of cellular, genetic, immunologic, metabolic, and endocrine pathways leading up to HCC which underscores the complexity of this condition. This article will provide an updated review of the pathogenic mechanisms leading from NASH to HCC as well as an exploration of the role of biomarkers and screening.

## 1. Introduction

Non-alcoholic steatohepatitis (NASH) is a chronic and progressive disease which leads to the accumulation of fatty deposits in the liver (steatosis) and subsequent inflammation [1]. NASH is a progression of non-alcoholic fatty liver disease (NAFLD) which is an umbrella term used to describe various forms of fatty liver disease in patients without alcohol consumption [2]. The prevalence of NALFD is increasing globally and is estimated to be about 25% [3]. However, this number is likely an underrepresentation, and the true prevalence of NALFD is much higher. Moreover, the prevalence of NAFLD among patients with diabetes is 56%, while the overall prevalence of NASH in diabetics is approximately 37% [4].

Globally, NASH has an incidence rate of 5.3 per 1000 and a hepatocellular carcinoma (HCC) incidence rate among patients diagnosed with NASH of 0.44 per 1000 persons [4]. Furthermore, in the US, NASH accounts for 18% of all HCC cases (an eightfold increase from 2002 to 2017) [5]. Being that NASH is on the rise, and given its significant risk for developing HCC, it is important to fully understand the mechanisms behind this progressive disease to derive more targeted therapies [6]. This article reviews the pathogenesis of NASH leading to HCC and emerging pathological concepts.

## 2. NASH and Liver Fibrosis

There are multifactorial insults that combine to induce cellular damage and activate cell death which leads to progressive liver disease. Persistence of these insults ultimately leads to activation of hepatic stellate cells, collagen deposition, and hepatic fibrogenesis (scar formation) [7]. Fibrosis in adults with noncirrhotic NASH is perisinusoidal, usually seen initially in acinar zone 3 [8]. Progressive scarring can develop followed by bridging fibrosis and cirrhosis [8].

In a systematic review and meta-analysis of paired liver biopsy studies, two distinct subsets of liver fibrosis were identified: rapid progressors (progression of stage 0 to bridging fibrosis or cirrhosis) and slow progressors (progression from stage 0 to stage 1 or 2 fibrosis) [9]. From this study, 20% of patients who developed interval fibrosis progression were classified as a rapid progressor. Furthermore, in patients with NASH and baseline F0 fibrosis, the annual fibrosis progression rate was on average 7.1 years to progress to stage 1, which was about half the average time for patients with NAFLD. From this meta-analysis, 40 (34.5%) patients developed progressive fibrosis, 45 (38.8%) patients remained stable, and 31 (26.7%) patients had improvement in fibrosis [9]. Thus, patients with NASH are at high risk for developing rapid liver disease. This poses serious public health implications for liver-related morbidity and mortality, especially in the absence of disease-modifying therapy.

## 3. Risk Factors for Fibrosis Progression

It is well known that NAFLD is strongly associated with metabolic syndrome, which includes obesity, dyslipidemia, hypertension, and increases the risk for developing type 2 diabetes melitus (T2DM) [10]. A 2020 cross-sectional study showed that progression of fibrosis stage in NASH was more likely to occur among obese patients [11]. In patients who progressed to the next stage or advanced stage fibrosis (141 patients), 59% had obesity, 57% had either hypertension, dyslipidemia, T2DM, or cardiovascular diseases.

The progression of NASH is less predictable than other forms of chronic liver disease. Lifestyle changes including weight reduction, physical exercise, and nutrient composition is vital to slowing the progression of liver fibrosis [12]. A mouse study compared metabolic and histological effects of a diet on the basis of composition and showed that a fast-food diet (high cholesterol, high saturated fat, and high fructose) administered for 6 months led to features of metabolic syndrome and NASH with progressive fibrosis [13]. In a study where 13 individuals were subjected to high-calorie fast-food meals, elevated serum alanine aminotransferase (ALT) levels and increased steatosis were found within a period of 4 weeks [14]. Additionally, Wei et al. demonstrated that fibrotic response was driven by high dietary fat. In C57Bl/6J mice models, choline-deficient, amino acid-defined (CDAA) diets with increasing fat content (10–60% by calories) were associated with steatohepatitis with robust fibrosis and ductular proliferation that progressed to cirrhosis and HCC within 24 weeks. [15]. However, Pompili et al. showed that a high carbohydrate–low fat diet was equally as harmful as a high fat diet alone [16].

These differences in diet may account for different rates of progressive liver disease. However, the current literature appears to indicate that carbohydrates (such as fructose) act in a synergistic fashion with diets rich in fat content (i.e., Western diet) to increase liver lipid accumulation, induce inflammation, and fibrosis [17].

This effect of diet in combination with oxidative stress was further exacerbated with long-term liver X receptor (LXR) agonist stimulation/expression [18]. LXR is an oxysterol-activated nuclear receptor involved in the control of major metabolic pathways for cholesterol homeostasis and lipogenesis where its expression has been correlated with intrahepatic inflammation and fibrosis [19].

Conversely, Schuppan et al. highlighted the role of lifestyle changes, such as weight loss, physical exercise, and a healthier nutrient composition on inflammation and fibrosis in NASH [12]. It demonstrated that an intensive weight loss program in 293 patients with NASH, 261 of whom were biopsied after 52 weeks, led to resolution of NASH in 25%, and reduced fibrosis in 19%. In those patients who lost ≥10% of body weight, NASH resolved in 90% and fibrosis improved in 45% [13]. Follow up studies on morbidly obese patients that underwent bariatric surgery demonstrated that the extent of weight loss can also correlate with the degree of resolution of NASH and even fibrosis regression [12]. Overall, these studies highlight the contribution of dietary composition and body weight in the development or regression of NASH and progressive fibrosis.

Of note, while NAFLD/NASH is defined by the absence of alcohol, however, a large proportion of patients consume mild to moderate amounts of alcohol. In a longitudinal study of 285 participants, Ajmera et al. reported that modest alcohol use was associated with less improvement in steatosis and level of aspartate transaminase, as well as lower odds of NASH resolution, compared with no use of alcohol [20]. However, a review of the literature has demonstrated conflicting results [21].

## 4. From “Double-Hit” to “Multi-Hit”

The first modern mechanism of NASH transformation to HCC was thought to have occurred via a “two-hit hypothesis” [22,23,24]. Initially, patients with obesity and/or T2DM develop insulin resistance [25]. Animal models have shown that high fat diets induce obesity-related insulin resistance and the release of inflammatory signals via toll-like receptor (TLR4) and nuclear factor kappa-light-chain-enhancer of activated B cells (NF-κB) pathways [26]. Chronic insulinemia impairs skeletal muscle and hepatic insulin signaling which promotes hepatic steatosis [27]. Moreover, excessive amounts of free fatty acids are produced from insulin-resistant adipose tissues via lipolysis. This creates a perpetual cycle of insulin resistance, accumulation of fatty acid metabolites, and steatosis [28].

The first hit is the development of steatosis, followed by lipid peroxidation caused by oxidation and inflammation of the liver, leading to necroinflammation and fibrosis, and ultimately HCC. Peroxidation of lipids found in cell membranes may lead to necrosis and megamitochondria [29]. The end products of this process include 4-hydroxynonenal and malondialdehyde (MDA) which activates hepatic stellate cells. Hepatic stellate cells are primarily capable of collagen production which crosslinks cytokeratins to produce Mallory bodies as well as promote chemotaxis of neutrophils [30,31,32]. MDA is also capable of inflammation by activating NF-κB which is a cell mediator in the expression of proinflammatory cytokines and adhesion molecules such as TNF-a, IL-8, intercellular adhesion molecule 1, and E-selectin [33,34].

However, many patients with steatosis never progress to fibrosis. This suggests that in addition to the “first hit,” a “second hit” is needed for the development of necroinflammation. Potential sources for this “second hit” include increased expression of CYP2E1 which can generate free radicals [35]. This inductive process is mediated in non-alcoholics by ketones and fatty acids (i.e., high fat diet) [36]. In patients with obesity and steatosis, the progression to fibrosis is accelerated by rapid weight loss during dieting, intestinal bypass surgery, surgical stress, alcohol intake, and T2DM, all of which increases free fatty acids in the liver.

The high concentration of free fatty acids in the liver provides a source of oxidative stress by peroxisomal b-oxidation which leads to the production of hydrogen peroxide. In the presence of iron, highly reactive hydroxyl radicals are released which contributes to mitochondrial damage and the precipitation of NASH/liver fibrosis [37,38,39,40]. This source of oxidative stress is required for initiating enough lipid peroxidation to overcome normal cellular defense mechanisms and produce necroinflammation [41,42].

Furthermore, diets with unbalanced polyunsaturated fatty acids (PUFA) have been linked to NALFD. Long term consumption of a Western diet which is high in saturated fat, omega-6 fatty acids and sugar (especially fructose), while deficient in omega-3 fatty acids, contributes to the development and progression of NAFLD [43]. Lower ratios of omega-6 to omega-3 have been shown to have a direct effect on Wnt signaling, decreasing expression of pro-inflammatory genes, and exhibiting less liver injury [44].

Diets high in sugars have been implicated in liver tumorigenesis. Qiao et al. reported that glucose induced advanced glycosylation end product-specific receptor (AGER) which was critical for liver tumorigenesis. Hyperglycemia appears to support cell proliferation, colony-formation capacity, and in vivo tumor growth while inhibiting apoptosis in HCC cells as well as other cancer types [45]. Furthermore, a follow-up study demonstrated that inadequate maintenance of blood glucose in patients with T2DM was a significant risk factor for HCC recurrence [46].

**Figure 1:**
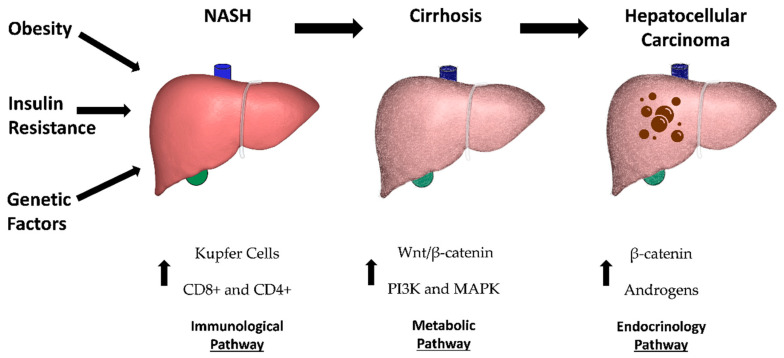
Risk factors and proposed mechanisms for non-alcohol steatosis (NASH) progression to hepatocellular carcinoma (HCC).

While still highly popularized, the simplicity of this hypothesis undermines the complex nature of NASH to HCC carcinogenesis. Further research has been done which has suggested a “multi-hit” or “multi-parallel hit” theory that takes into consideration several theorized models, all of which play a role in the oncogenesis of HCC [23]. There are currently four additional proposed prongs to the multi-hit theory which includes genetics, immunologic, metabolic, and endocrine pathways (Figure 1).

### 4.1. Genetic Mechanism

Genetic modifiers have been shown to play a role in the pathogenesis of liver fibrosis in NASH. Two genes have been well established with proposed mechanisms: patatin-like phospholipase domain-containing protein 3 (PNPLA3) and transmembrane 6 superfamily member 2 (TM6SF2) [47,48]. PNPLA3 is an adiponutrin that is found in intracellular membrane fractions within hepatocytes and has lipolytic activity [49]. PNLPA3 has been established as having a direct association with hepatic steatosis, steatohepatitis, elevated plasma liver enzyme levels, hepatic fibrosis, and cirrhosis [50,51,52,53]. PNLPA3 also has a role in retinol metabolism and production by hepatic cells, which likely has a role in inflammation of the liver [54]. It has been observed in mice and human models that diets high in fat and carbohydrate create an anabolic milieu thereby upregulating PNPLA3 [55,56,57,58]. Perttilä et al. showed that glucose exerts an indirect effect on PNPLA3 via carbohydrate response element-binding protein (ChREBP) in hepatocyte [59]. PNPLA3 expression is upregulated by both insulin and glucose [60].

Knockout PNPLA3 mice models on a high fat diet have reduced liver fat content despite the high fat diet. This suggests that the PNPLA3 gene plays a profound role in lipid esterification and lipogenesis in the liver [61,62]. In a 2018 study, it was shown that PNPLA3 mediates the transfer of polyunsaturated fatty acids from triglycerides to phospholipids in hepatocytes [63]. In other words, elevated PNPLA3 protein levels lead to lipogenesis thereby increasing the amount of fat content within the liver. Conversely, reducing *PNPLA3* expression levels could potentially attenuate its negative effect on hepatic lipolysis [64]. However, some studies have cited contrary evidence [65]. This suggest that previous research may have unknown confounding variables such as up or down stream regulators and compensatory mechanisms which need to be further defined.

On the other hand, TM6SF2 is believed to function as a lipid transporter which may interact with proteins involved in intestinal absorption [66]. Studies using confocal microscopy have demonstrated localization of GFP tagged TM6SF2 to the endoplasmic reticulum and Golgi compartment. Knockout TM6SF2 in-vitro experiments showed reduced secretion of triglyceride-rich lipoproteins and Apo-B [67]. As a result, there was increased lipid droplet number and size. Alternatively, overexpression of TM6SF2 showed decreased number and size of lipid droplets. In vivo studies further support this phenotypic expression of TM6SF2. Overall, these studies suggest that TM6SF2 regulated lipid influx and efflux depending on its deletion, mutation, or overexpression.

Individuals carrying the minor (T) allele of TM6SF2 rs58542926 (167K) appear prone to developing NAFLD with advanced fibrosis and so are more likely to experience liver-related disease rather than cardiovascular morbidity [68]. Alternatively, carriage of the C-allele is associated with dyslipidemia and cardiovascular disease with a lower incidence of NASH [69]. Nevertheless, further studies with associated human genetic variants will greatly inform our understanding of the pathophysiology and interrelationship between NASH and stages of fibrosis.

Dysregulation in the synthesis of fatty acids is associated with NASH. Prior studies have shown that FADS1 is a regulator of hepatic lipid composition [70]. However, Chiappini et al. showed concomitant increase in saturated and unsaturated long chain fatty acids (LCFA) with significant decrease in polyunsaturated very long chain fatty acids (VLCFA) from impaired activity of FADS1, independent of obesity [71]. Such metabolic alterations may generate broad effects since LCFA is a substrate for the synthesis of eicosanoids and phospholipids, and a precursor for the synthesis of lipid signaling pro-inflammatory molecules [72,73,74].

It is important to note the role of microRNA (miRNA). Prior studies have shown that miRNA is associated with NASH [75]. There has also been miRNA that have correlated with distinct pathways which leads to the oncogenesis of hepatocellular carcinoma [76]. When cross-referencing both miRNA for NASH and HCC, no distinct miRNA was found, but common pathways have been identified [77]. For example, in mice models there are distinct miRNA associated with the phosphatase and tensin homolog (PTEN) protein in the NASH and HCC pathway [78]. In mice models, those that were PTEN deficient developed steatosis, hepatomegaly, and HCC [79]. Mutual pathways leading to a disease state for both NASH and HCC further develop the idea that there is not only a link but the possibility for association or progression of one disease state to the next.

While there are specific genetic mutations that have clinically significate roles in the oncogenesis of HCC, there is another component that cannot be overlooked—DNA damage repair and response. DNA damage is a daily occurrence within the human genome from various internal and external processes that persistently lead to DNA damage. In the setting of improper repair with inadequate or overstimulated response there is potential for HCC to develop.

In various mice models for studying NASH, it has been shown that as oxidative damage increases, DNA repair enzymes decrease in direct proportions. This logically suggests that DNA repair deficiency can lead to NASH and potentially HCC. This has a more profound impact clinically as patients with inherited or acquired DNA repair enzyme dysfunction leads to susceptibility to NASH and progression to HCC [80]. For example, ataxia-telangiectasia mutated (ATM) kinase deficiency leads to a syndrome known as ataxia-telangiectasia. The ATM is known for its contribution to DNA genomic stability and role in DNA repair [81,82,83]. When ATM is no longer functional it has been seen in mice models that develop steatosis and fibrosis and as established is well known inciting factor for the development of HCC [84]. In study by Schults et al., the authors were able to evaluate liver biopsy specimens and determine that the specimens that expressed a high degree of inflammation, also expressed a reduction in DNA damage repair, further supporting the hypothesis that DNA repair plays a role in HCC progression [85].

Another protein being currently studied in DNA-dependent protein kinase (DNA-PK) plays a role in the repair of DNA breaks, and interestingly plays a role in lipogenesis as well [86]. DNA-PK is interesting—while in ATM there is a downregulation leading to more DNA damage, in DNA-PK there is upregulation which leads to DNA damage. DNA-PK works when both strands in the DNA molecule break DNA-PK which forces the two broken strands back together in a process called non-homologues end joining [87]. Since DNA-PK does not correct for any missing nucleotides and sequences when recombining the strands, this leads to an error prone process. DNA-PK results in upregulation of DNA repair; as DNA-PK repairs, DNA is also prone to cause DNA error leading to DNA mutations and unstable cells thereby leading to HCC [88,89]. There are studies being done that are evaluating whether patients with NASH-associated HCC with DNA-PK upregulation are found to have a worse response to localized chemoembolization therapy [90]. With this information, there are current studies being done to determine targeted therapy to downregulate DNA-PK activity in patients with HCC, with preliminary data with in vivo and in vitro studies suggesting suppression of DNA-PK as a promising therapy [91].

While we have described the major and established genetic players in NASH and HCC, other genes have been identified which require further investigation. Desterke et al. identified 25 genes that are commonly found to be dysregulated during steatosis progression to NASH and cancer [92]. Furthermore, Nwosu et al. identified 634 metabolic-related genes mostly (*n* = 350) downregulated and involved in physiologic hepatocyte metabolic functions (e.g., xenobiotic, fatty acid, and amino acid metabolism) and upregulated (*n* = 284) in glycolysis, pentose phosphate pathway, nucleotide biosynthesis, tricarboxylic acid cycle, oxidative phosphorylation, proton transport, membrane lipid, and glycan metabolism [93]. These genes are potentially relevant targets for clinical studies, however, they require additional investigation.

### 4.2. WNT/β-Catenin Signaling

In those patients that display insulin resistance and hyperinsulinemia, there is an increase in serum insulin and insulin-like growth factor. Insulin and insulin growth factor-1 (IGF-1) bind to respective receptors which leads to the activation of two distinct pathways, downstream PI3K and MAPK pathways [94,95]. It has been established that PI3K and MAPK pathways play a role in oncogenesis by induction of cell proliferation and inhibition of apoptosis. Furthermore, downstream of MAPK leads to activation of Wnt/β-catenin which leads to fibrosis and HCC [96].

Interestingly, mice with wild-type or mutant β-catenin are not able to develop spontaneous liver cancer [97,98]. As a result, inactivation of β-catenin alone is not sufficient to develop HCC. However, β-catenin activation may work in tandem with other oncogenic pathways including insulin/IGF-1/IRS-1/MAPK, H-RAS, MET, AKT and chemicals to promote HCC [99,100,101]. Other epigenetic modifications may also contribute to the activation of Wnt/β-catenin signaling, including hypermethylation of Wnt antagonists, deacetylation of histones in the AXIN2 promoter, and downregulation of microRNAs negatively regulating Wnt/β-catenin signaling.

Lastly, while not fully understood, it is important to note the tumor microenvironment and the role of autophagy. Autophagy is an evolutionary conservative intracellular mechanism involved in diverse liver physiology and pathology. Unlike other tissues or organs, autophagy in the liver leads to synthesis of adipose tissue which leads to NASH [102,103,104]. Activation of autophagy inhibited proliferation of HepG2 cells in blocking GOC3/Wnt/B-catenin signaling [105,106]. Furthermore, autophagy can induce Monocarboxylate transporter 1 (MCT1) expression which is involved in lactic acid transport and H+ clearance in cancer cells, by activating Wnt/β-catenin signaling. Overall, this process leads to metastasis and glycolysis in HCC cells [107]. In addition, pharmacological inhibition of v-ATPase/autophagy blocks HCC cell proliferation (Table 1).

Another important aspect in the Wnt pathway is the role of exosomes. Prior studies have shown an association between exosomes and Wnt signaling in the migration of gastric and colon cancer cells [108,109,110,111]. However, it is unclear whether Wnt signaling plays a critical role in the invasion and migration of liver cancer and warrants further investigation.

### 4.3. Immunological Pathway

The next prong that needs to be evaluated is the immune response in the setting of liver injury from reactive oxygen species (ROS). When ROS disrupt the mitochondria and lipid peroxidation, this leads to a release of inflammatory markers including tumor necrosis factor-alpha (TNF-α), interleukin-6 (IL-6), leptin, and adiponectin [91]. In patients with insulin resistance, there are reduced levels of adiponectin which directly leads to an increase in angiogenesis and decrease in apoptosis, thereby increasing the risk of NASH conversion to HCC [112,113].

The role of the adaptive immune system is still very unclear, but several mouse models have been developed. Two studies have shown that CD8+ and CD4+ T-lymphocytes are involved in liver damage and subsequent oncogenesis [114,115]. Furthermore, as more liver damage occurs from steatosis, Kupfer Cells are activated which recruit other cells thereby contributing to NASH [116,117]. On the other hand, natural killer cells are activated by ligands and cytokines (CD107a and cytokine production of IFN-γ, TGF-β and IL-10) have been associated with a protective role in liver disease [116,118].

Mice that were induced to have macrovesicular steatosis, necro-inflammation and fibrosis in the liver showed increase numbers of NK cells in the liver but decreased in the spleen [119]. In NK cell-deficient Nfil3-/- mice, similar levels of TG and macrovesicular steatosis was observed, but more inflammatory infiltration and increased collagen deposition were found in the liver. Furthermore, the depletion of NK cells caused a significant increase in the infiltration of monocyte-derived macrophages [120]. Overall, the data suggest that intrahepatic NK cells play a protective role against the fibrosis progression in NASH [121].

Sonic Hedgehog genes have been shown to play a major role in liver repair through the mobilization of hepatic precursor cells [122]. In studies where SHH signaling is impaired, improper liver repair leads to the conversion of NASH to HCC [101]. Further studies are needed to define the pathophysiological role of the adaptive immune system in the development and progression of NASH into HCC.

### 4.4. Endocrine Pathway

**Figure 2:**
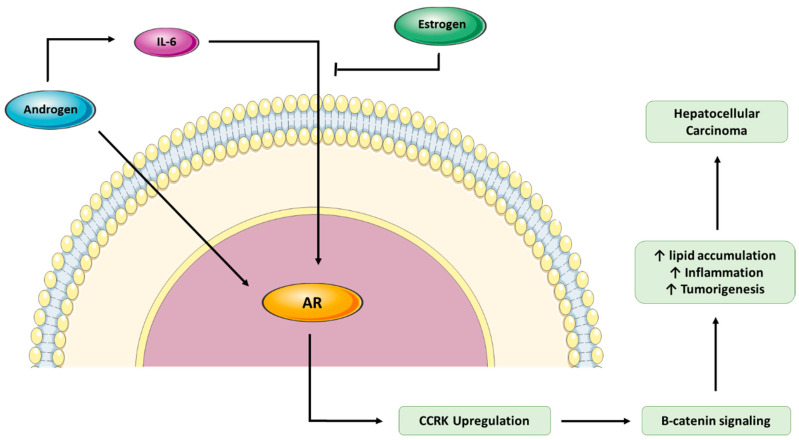
Role of androgens and estrogens in tumorigenesis. AR: androgen receptor, CCRK: cell cycle-related kinase.

The final prong in this multi-hit model for the oncogenesis of NASH to HCC is the endocrine pathway. As previously reported above, middle-age men had the highest incidence of NASH to HCC, which derives the question of whether gender-specific hormones play a role in NASH and HCC (Figure 2). Reports have shown that patients with hypothyroidism might lead to NASH, cirrhosis and potentially liver cancer via the development of hyperlipidemia and obesity [123]. Similarly, patients with growth hormone deficiency have a metabolic-syndrome-like phenotype that is also associated with the development of NASH [123]. Polycystic ovary syndrome is a common endocrine disorder that is often associated with insulin resistance, the metabolic syndrome, altered levels of liver enzymes and the development of NASH [123].

In addition, adrenal failure is increasingly reported in patients with end-stage liver disease and in patients who have received a liver transplant, which suggests a bidirectional relationship between liver and endocrine functions [123]. Recent findings support a role of dehydroepiandrosterone sulfate deficiency in the development of advanced NASH [123]. In an early study from 1995, it was proposed that male androgens promote HCC compared to estrogen which suppresses the development of HCC [124]. In 2012 it was determined that androgens stimulate the transcription of cell cycle-related kinase (CCRK) which upregulates β-catenin [125]. Murine studies with knockout CCRK showed suppressed hepatic lipid accumulation, inflammation and tumorigenicity in NASH and HCC models. Mechanistically, obesity-induced pro-inflammatory and upregulated CCRK expression led to the activation of the mTORC1 pathway crucial for lipid/glucose homeostasis, immunosuppression, and tumorigenesis [126]. The data suggest that CCRK functions as a major signaling hub in obesity-associated hepatic oncogenesis [123].

## 5. GALAD Score and Role of Biomarkers

The GALAD score was originally curated by Johnson et al. with the goal of establishing a quantitative model for the development of HCC in chronic liver disease patients [127]. The score measures five variables including gender, age, α fetoprotein, AFP-L3%, and des-carboxy-prothrombin. In a validation study in 2016, the GALAD score had a sensitivity of 85.6% and a specificity of 93.3%. The GALAD score showed a superior detection rate of early-stage HCC and AFP-negative tumors [128]. In patients with NASH, the GALAD score has also been shown to detect HCC independent of cirrhosis or stage of HCC with a sensitivity of 91.2% and a specificity of 95.2% [129].

Other novel biomarkers have become potential candidates for HCC screening. In an animal model study, two potential markers—serum glycoprotein osteopontin and dickkopf-1—were determined [130]. Another potential marker is circulating tumor DNA (ctDNA) which can be used to determine if specific genetic mutations have occurred [131]. The ctDNA would be for common pathways involved in HCC oncogenesis such as p53 signaling, the Wnt–β-catenin pathway, chromatin remodeling, responses to oxidative stress (for example, KEAP1 and NFE2L2) or telomere maintenance pathways [96]. Additionally, circulating miR-122, (a liver-specific miRNA) responsible for liver homeostasis, has emerged as a sensitive biomarker for liver injury [132]. A few genes have also been discovered that can become hypomethylated or hypermethylated and can be detected via ctDNA of these genes; there are three currently proposed in the involvement of HCC oncogenesis: GSTP1, RASSF1 and LINE [133,134,135] (Table 2).

GSTP1 has been negatively correlated with tumor size and serum alpha-fetoprotein (AFP) in HCC patients while higher GSTP1 levels have been associated with longer overall survival and better prognosis [136]. RASSF1 has been shown to be hypermethylated and can be used as a biomarker to distinguish HCC from other liver neoplasms [137]. LINE-1 hypomethylation was shown to be associated with shorter overall survival [137].

Furthermore, the emergence of machine learning-based approaches opens a new avenue for early diagnosis of NASH and HCC. Chiappini et al. demonstrated using mouse models that machine learning based on prediction analysis of microarrays was able to identify lipid signatures related to NASH (21 lipids out of 149 in the liver and 14 lipids out of 155 in the serum) [138]. Further clinical and cost-effective studies are needed to validate these potential biomarkers in the diagnosis and prognosis of this condition.

## 6. Screening and Cost

NAFLD is a global epidemic and is becoming the leading cause of chronic liver disease worldwide, with an estimated prevalence of approximately 20–30% in Western populations [3,139]. However, this estimate is largely underrepresented, and the true prevalence is likely higher. As the rates of T2DM and obesity increase worldwide, it is expected that NAFLD will become more common. NAFLD-related cirrhosis is currently the third most common indication, after hepatitis C and alcoholic cirrhosis, and is anticipated to become the leading indication for liver transplantation in the USA within the next one to two decades [140]. However, given the current trajectory and scale of this epidemic, NAFLD/NASH threatens the future of liver transplantations due to a lack of viable organs to meet the demand [141,142].

Additionally, long-term mortality studies in NAFLD patients during 28 years of follow-up showed a 69% (standardized mortality ratio (SMR) = 1.69; 95% confidence interval [CI], 1.24–2.25) higher risk of death compared to a matched cohort [143]. The main factor leading to early mortality and morbidity in NAFLD patients is the development of advanced fibrosis [144]. Hence, it is crucial to investigate and consider this condition during its early stages prior to fibrotic changes. Identifying patients with NASH in earlier stages of fibrosis is supported by several smaller studies and a recent meta-analysis that demonstrated a 1.41-, 9.57-, 16.69- and 42.3-fold increase in liver-related mortality, in subjects with stage 1, 2, 3 and 4 fibrosis, respectively [145].

Given the increased mortality rate associated with advanced fibrosis, a form of practical routine screening of NAFLD is necessary to improve patient outcomes. The standard for definitive diagnosis of NAFLD/NASH is performed by liver biopsy to assess for steatosis, inflammation, and fibrosis. However, the invasiveness of liver biopsy prevents its routine use, hence, better and less invasive diagnostic modalities are needed to detect NAFLD/NASH. The use of biomarkers and non-invasive assessments of liver disease can be initially used in screening for NAFLD, NASH, and early fibrosis, as they have been shown to be reliable predictors of liver-related outcomes and overall mortality [146].

Currently, there is no standard medical therapy with proven efficacy available for treating NAFLD/NASH [147]. Most patients are advised to engaged in lifestyle and dietary modifications. Due to its wide prevalence and rising incidence, the diagnosis and treatment of NASH is costly. In the US, the annual economic burden of NAFLD is estimated to be USD 103 billion, where NASH accounted for about USD 15.4 billion [148]. Without taking into consideration the cost of treatment, the 10-year burden is estimated to surpass USD 1 trillion. Thus, the importance of consensus in screening and treatment guidelines is needed. However, no guidelines exist among professional organizations to recommend screening.

## 7. Summary

NASH is a growing global epidemic and public health threat. Concomitantly, the incidence of HCC is projected to increase worldwide [120]. Our review shows several pathways by which NASH progresses to HCC. It underscores the complexity of this disease and the effects of cellular, genetic, immunologic, metabolic, and endocrine pathways. However, over the last decade there has been progress in identifying pharmacological targets as well as potential biomarkers. However, the rapidity at which this disease is rising supersedes scientific momentum. As a result, further work is needed if we are to understand and develop methods for decreasing steatosis, rates of fibrosis, and HCC.

## Figures and Tables

**Table 1:** Pathways contributing to the development of hepatocellular carcinoma (HCC) from non-alcoholic steatohepatitis (NASH).

Pathway	Primary Mechanism
Cellular	Steatosis followed by lipid peroxidation
Genetic	Elevated PNPLA3 protein levels facilitate lipogenesis Decreased TM6SF2 levels reduce lipid efflux, increase lipid droplet number and size
Immunologic	Cytokine release recruit Kupfer cells and contribute to NASH Decreased NK cells associated with infiltration of monocyte-derived macrophages
Metabolic	Insulin and IGF-1 signaling associated with PI3K and MAPK activation of Wnt/β-catenin along with epigenetic modifications facilitate fibrosis
Endocrine	Androgens stimulate transcription of cell cycle-related kinase (CCRK) which upregulate β-catenin

**Table 2:** Possible biomarkers used for the detection of hepatocellular carcinoma (HCC).

Biomarker	Role in HCC Development
Osteopontin	Glycoprotein of the extracellular matrix, overly expressed in HCC
Dickkopf-1	Inhibitor of Wnt/β-catenin signaling, overly expressed in HCC
miR-122	Marker of liver injury; suppressed in HCC
GSTP1	Negatively correlated with tumor size and overall survival
RASSF1	Positively correlated with longer overall survival and better prognosis
LINE-1	Hypomethylation associated with shorter overall survival
